# To Open the Eustachian Tube

**Published:** 1896-02

**Authors:** 


### To Open the Eustachian Tube.

Recognizing the danger of allowing the uninitiated to use the Politzer air bag for this purpose, Dr. N. R. Gordon, of Springfield, Ill., thus describes (*Medical Brief*), a method original with him:

"Take into the mouth a full sup of water, grasp the nose tightly with the thumb and finger, try to blow air out through the nose and at the same time throw the head far back, so the water will run into the back part of the mouth and force upward the velum palati, then swallow the water and the air will gently open the eustachian tubes. This act can be repeated frequently without injury; it is nature's own way, only in an exaggerated form, for opening the tubes.

A MIXTURE of chloroform (ten parts), ether (fifteen parts), and menthol (one part), used as a spray, is recommended as an excellent and prompt means for obtaining local anæsthesia, lasting for about five minutes.—*Boston Med. and Surg. Journal.*

